# Physiological Responses in a Variable Environment: Relationships between Metabolism, Hsp and Thermotolerance in an Intertidal-Subtidal Species

**DOI:** 10.1371/journal.pone.0026446

**Published:** 2011-10-17

**Authors:** Yun-wei Dong, Shan-shan Yu, Qing-lin Wang, Shuang-lin Dong

**Affiliations:** 1 State Key Laboratory of Marine Environmental Science, College of Oceanography and Environmental Science, Xiamen University, Xiamen, Fujian Province, People's Republic of China; 2 The Key Laboratory of Mariculture, Ministry of Education, Fisheries College, Ocean University of China, Qingdao, Shandong Province, People's Republic of China; Argonne National Laboratory, United States of America

## Abstract

Physiological responses to temperature reflect the evolutionary adaptations of organisms to their thermal environment and the capability of animals to tolerate thermal stress. Contrary to conventional metabolism theory, increasing environmental temperatures have been shown to reduce metabolic rate in rocky–eulittoral-fringe species inhabiting highly variable environments, possibly as a strategy for energy conservation. To study the physiological adaptations of an intertidal-subtidal species to the extreme and unpredictable heat stress of the intertidal zone, oxygen consumption rate and heat shock protein expression were quantified in the sea cucumber *Apostichopus japonicus.* Using simulate natural temperatures, the relationship between temperature, physiological performance (oxygen consumption and heat shock proteins) and thermotolerance were assessed. Depression of oxygen consumption rate and upregulation of heat shock protein genes (*hsps*) occurred in sequence when ambient temperature was increased from 24 to 30°C. Large-scale mortality of the sea cucumber occurred when temperatures rose beyond 30°C, suggesting that the upregulation of heat shock proteins and mortality are closely related to the depression of aerobic metabolism, a phenomenon that is in line with the concept of oxygen- and capacity-limited thermal tolerance (OCLTT). The physiologically-related thermotolerance of this sea cucumber should be an adaptation to its local environment.

## Introduction

Physiological adaptations are important for animals to cope with changing environmental conditions and are considered to play a major role in determining which species will be “winners” or “losers” under scenarios of global climate change [1], [2]. The concept of oxygen- and capacity-limited thermal tolerance (OCLTT) introduced by Pörtner provides a framework to link an organism's physiology with its ecological landscape, and indicates that a mismatch in the demand for oxygen and the capacity to supply tissues with oxygen is the first mechanism which limits a species tolerance of thermal extremes [3], [4]. When temperature exceeds the pejus temperature (T_p_, which is the limit of optimum haemolymph oxygenation), a drop in aerobic scope arises due to an increasingly reduced capacity of the circulatory and ventilatory systems. Excessive warming beyond this limit eventually leads to a complete loss of aerobic functionality at the high critical threshold temperature (T_c_), which is concomitant with mitochondrial metabolism switching to an anaerobic mode. The theory has been derived from studies of marine fishes and invertebrates from various climatic regions [3]–[5]. However, it remains unclear how generally the OCLTT can be applied to all taxonomic and ecological situations. The theory has, for example, received limited support in the case of air-breathing ectothermic animals [6], [7].

In highly variable environments, some animals can utilize metabolic depression, anaerobic energy production, and stress protection mechanisms to provide protection against extreme temperatures [4]–[8]. In the intertidal zone, organisms can spend part of their lives immersed in stable and benign seawater and the remaining time in extreme and arid conditions during emersion. Therefore, the relationship between temperature and physiological performances (metabolism and stress protein expression pattern) of animals living in the intertidal zone can therefore provide a suitable model to elucidate their physiological adaptation to temperature fluctuation and hence to changing temperatures appropriate to global change in the highly variable thermal environment.

The sea cucumber *Apostichopus japonicus* (Selenka) is widely distributed along the Asian coast from 35°N to 44°N in Russia, China, Japan and Korea [9]–[10]. Juveniles of this species prefer to live in the low intertidal zone (+0.4 m above Chart Datum) on rocky substrates [11]. Young sea cucumbers, over 2 years old, often leave the intertidal zone (their nursery habitat) before winter and migrate to the subtidal zone (the adult habitat) [12]. The reasons for this migration are still, however, unclear and need further investigation [13]. In its natural habitat, this sea cucumber encounters frequent temperature variations during the tidal cycle, which become extreme during low spring tides, especially in the summer when the body temperature of the sea cucumber can increase rapidly from 20°C to over 30°C (Dong and Meng, unpublished data).

*Apostichopus japonicus* is a typical ectothermic species in which body temperature is largely determined by environmental thermal conditions, and temperature plays an important role in growth and other physiological performances [14]–[17]. Growth is closely related to temperature, and is optimized at temperatures between 12°C –18°C [18]. This species can, however, survive the water temperature of 0°C, as well as temperatures above 30°C during hot summer months. The lethal temperature of 50% of the sea cucumbers (TL_50_) after 2-hour exposure is ∼ 31°C [19]. The oxygen consumption of *A. japonicus* is also positively temperature-dependent within the range of 10–25°C, and decreases above 25°C [14]–[18]. When sea cucumbers are kept at 26°C for 2 weeks, they enter a state of aestivation which involves gut tract degeneration, weight loss and metabolic rate depression [20]. Furthermore, temperature fluctuation has been found to significantly affect growth, metabolism and heat shock protein expression in this species [15], [16].

In the present study, we hypothesize that the thermal tolerance of *A. japonicus* is closely related to its capacity for oxygen consumption and that of expression of heat shock proteins. Oxygen consumption and heat shock protein expression were determined for a series of thermal regimes that simulate natural temperature regimes. This study therefore contributes to our understanding of the physiological adaptation of these intertidal animals to temperature, and to determine whether these species conform to the concept of oxygen- and capacity-limited thermal tolerance.

## Results

### Metabolic performance

**Figure 1:**
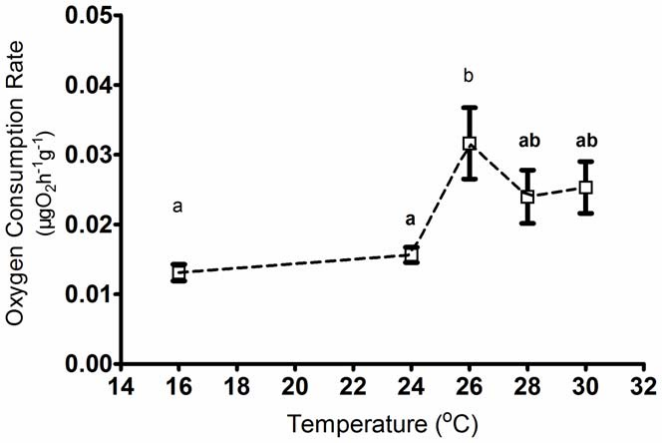
The peak values of oxygen consumption in different temperature treatments (24, 26, 28, and 30°C). Different letters indicate significant difference (*P*<0.05).

Oxygen consumption rate (OCR) was closely related to temperature (Figure S1). There were significant differences in OCR at different time points (Two-way ANOVA, time points, F _(11, 295)_  = 10.357, *P*<0.001) and temperature treatments (F _(4, 295)_  = 16.737, *P*<0.001). At 16°C for 24 h, sea cucumbers had a relatively stable metabolic rate with slight daily variation (between 0.0127∼0.0177 µg O_2_ h^−1^ g^−1^, One-way ANOVA, F _(10, 54)_ = 1.407, *P* = 0.209). When temperature increased from 16°C to 24°C, OCR increased from 0.011 to 0.156 µg O_2_ h^−1^ g^−1^, and there was significant difference among different time points (One-way ANOVA, F_(11, 59)_ = 2.889, *P* = 0.005). When temperature increased from 16°C to 26°C, 28°C and 30°C, OCR increased to 0.032 µg O_2_ h^−1^ g^−1^, 0.023 µg O_2_ h^−1^ g^−1^ and 0.025 µg O_2_ h^−1^ g^−1^, respectively. The peak values of OCR in the different temperature treatments (F24, F26, F28, F30) occurred at the same time (18:00), and they were significantly different among treatments (One-way ANOVA, F _(4, 24)_ = 0.017, *P* = 0.017). The peak values of OCR increased from F24 to F26, and showed a decline trend in F28. The peak value of OCR in F26 was significantly higher than those in F24 (Duncan test, Figure 1).

### Heat shock response

The expression of *hsp70* in the three tissues (intestine, respiratory tree and body wall) showed similar trends with variation of temperature. In the 24-hour temperature cycle, the expression of *hsp70* increased with the increase of temperature, and decreased immediately when water temperature decreased (Figure S2).

**Figure 2:**
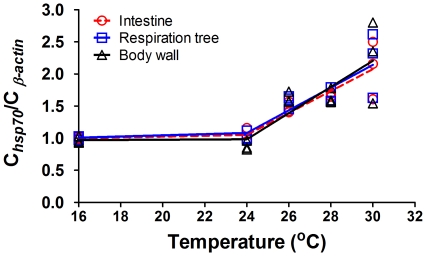
The two-stage segmental regression of the peak values of*hsp70* and temperature of sea cucumber, *Apostichopus japonicus* in intestine, respiratory trees and body wall.

The peak value of *hsp70* in F24 treatment occurred at 14:00 (intestine, C*_hsp70_*/C_β-actin_ = 1.186±0.110; respiratory tree, C*_hsp70_*/C_β-actin_ = 1.070±0.061; body wall, C*_hsp70_*/C_β-actin_ = 0.923±0.076), at that time water temperature reached 24°C. In F26, F28, and F30, the peak values of *hsp70* occurred at 18:00, at that time the water temperature had been at the maximum temperature for 4 hours. The increase of the peak values of *hsp70* can be divided into two stages with temperature increase (Figure 2). In all the three tissues, *hsp70* levels increased slowly between 16 and 24°C and increased rapidly above 24°C until 30°C (Table S1).

The expression patterns of *hsp90*s were similar to those of *hsp70* (Figure S3, Figure S4). Levels of *hsp90*s increased with temperature and decreased when temperature was lowered. The peak values of *hsp90*s occurred at the maximum temperatures (at 14:00 or 18:00) in the 24-hour temperature cycle. In all three tissues, there were significant differences in expression of *hsp90a* at different time points (two-way ANOVA intestine, F _(4, 60)_ = 93.534, *P*<0.001; respiratory tree, F _(4, 60)_ = 93.534, *P*<0.001; body wall, F _(4, 60)_ = 16.679, *P*<0.001) and temperature treatments (intestine, F _(3, 60)_ = 89.191, *P*<0.001; respiratory tree, F _(3, 60)_ = 46.846, *P*<0.001; body wall, F _(3, 60)_ = 32.765, *P*<0.001). The expression of *hsp90b* showed a similar pattern and significant differences between the different time points (intestine, F _(4, 58)_ = 31.331 *P*<0.001; respiratory tree, F _(4, 60)_ = 58.372, *P*<0.001; body wall, F _(4, 60)_ = 11.022, *P*<0.001) and temperature treatments (intestine, F _(3,58)_ = 52.352, *P*<0.001; respiratory tree, F _(3, 60)_ = 29.916, *P*<0.001; body wall, F _(3, 60)_ = 27.463, *P*<0.001).

**Figure 3:**
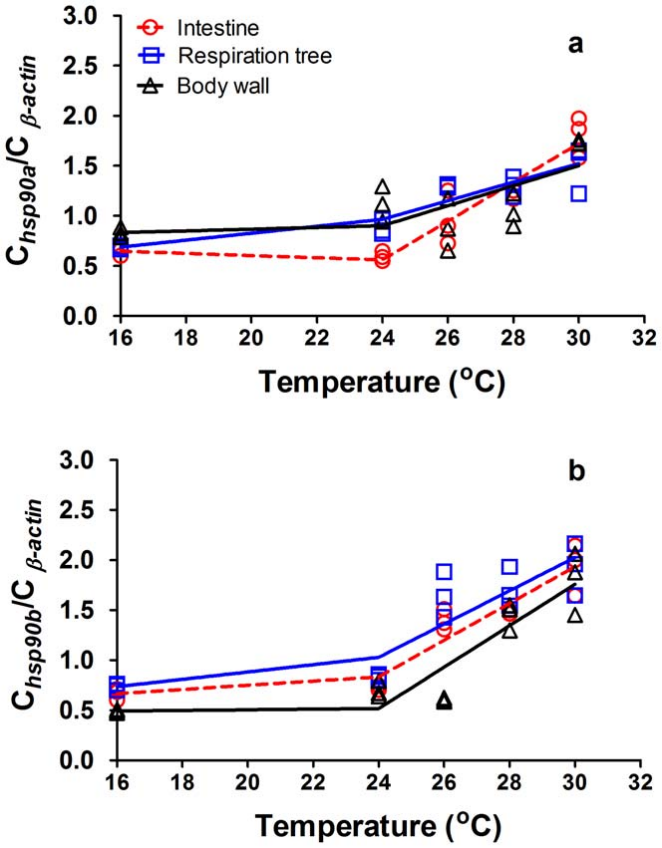
The two-stage segmental regressions of the peak values of (a) *hsp90a* and (b) *hsp90b* and temperature of sea cucumber *Apostichopus japonicus* in intestine, respiratory trees and body wall.

The peak values of *hsp90*s can also be expressed as two-stage segmental linear regressions (Table S1, Figure 3). Expression of *hsp90*s increased slowly until 24°C and then increased rapidly when temperature was over 24°C. In the intestine and respiratory tree tissues, the peak values of *hsp90*a levels at F26, F28 and F30 were significantly higher than at F24 and 16°C constant temperature (intestine: F _(4,14)_ = 30.890, *P*<0.001; respiratory tree: F _(4,14)_ = 36.521, *P*<0.001). In the body wall, the peak values of *hsp90a* at F30 were significantly higher than the peak values at F28, F26, F24 and at 16°C constant temperature (body wall: F _(4, 14)_ = 9,243, *P*<0.001). The peak values of *hsp90b* showed a similar trend to *hsp90a*, but those of *hsp90b* at F28 and F30 were significantly higher than those at F24 and at 16°C constant temperature exposure (intestine: F _(4,14)_  =  97.539, *P*<0.001; respiratory tree: F _(4,14)_  =  54.315, *P*<0.001; body wall: F _(4,14)_  =  83.563, *P*<0.001).

## Discussion

Oxygen consumption of the sea cucumber, *Apostichopus japonicus,* was temperature-dependent. Under a constant temperature of 16°C, OCR was stable and there was no significant daily variation. When temperature changed in a simulation of natural temperature conditions, a close relationship was found between OCR and temperature, indicating that metabolism of the sea cucumber is very sensitive to environmental temperatures [see previous studies 14, 16, 17]. In each thermal regime, the maximum values of oxygen consumption rate occurred at the designated maximum temperature at 18:00. At that time, the sea cucumbers had been maintained at the maximum temperatures for 4 h, and so the 2-h interval OCR measured at 18:00 could be regarded as the OCR at the maximum temperature. The peak values of OCR in each temperature regime increased with temperature until 26°C and then decreased, indicating that the aerobic metabolism of the sea cucumber (body weight, 10.43±0.49 g) is limited when temperatures rise beyond 26°C. This limitation on the capability of aerobic metabolism seems to be a most important factor in determining the thermotolerance of this species and its ability to survive high summer temperatures. When ambient temperatures rise beyond 26°C, anaerobic metabolism seemingly increases to support the additional energy required to counter the pending thermal stress. The transformation from aerobic to anaerobic metabolism and related changes of the cellular biochemical pathway [3], [21], [22] will affect the organism's thermal tolerance accordingly [23].

When heat shock is encountered, the upregulation of heat shock proteins occurred as a defensive mechanism in the sea cucumber [19], [20], [24]–[27]. In all the temperature regimes, the levels of *hsp*s (*hsp70*, *hsp90a* and *hsp90b*) of the sea cucumbers increased with an increase in temperature and *vice versa* in all three tissues, indicating a temperature-dependent expression of the three *hsp* genes at the transcriptional level. Overall, the maximum values of *hsp*s in each temperature treatment matched elevated metabolic performance as indicated by oxygen consumption. Comparison of the peak values of *hsp*s at the different simulated temperatures showed that the initial temperature for *hsp*s induction occurred at 26°C, and the levels of *hsp*s increased with warming from 26 to 30°C. These results further suggest that sea cucumbers reared at water temperatures beyond 26°C must suffer thermal stress.

**Figure 4:**
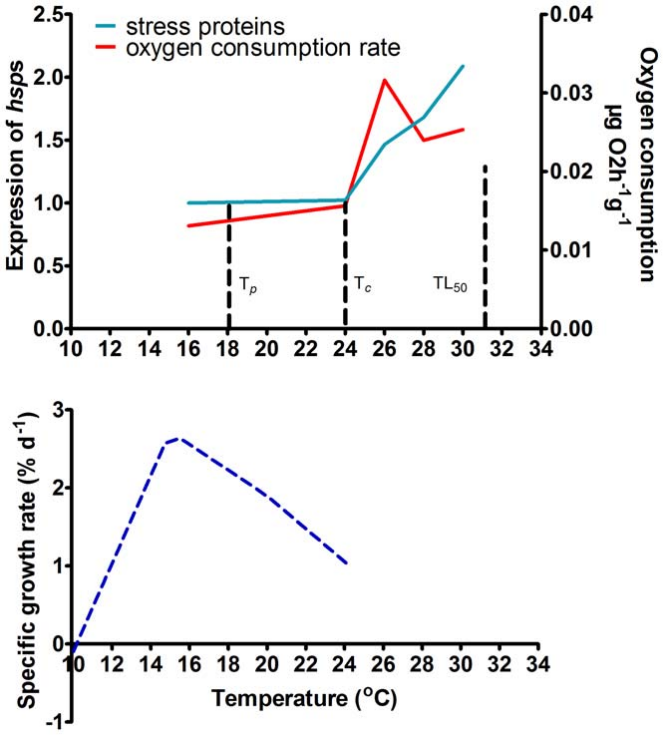
The variation trend of expression of *hsp*s, oxygen consumption rate and specific growth rate (SGR) with temperature increase in the sea cucumber *Apostichopus japonicus*. Pejus temperature (T_p_), which is the limit of optimum hemolymph oxygenation and therefore temperatures above this will result in a worsening of oxygen supply to the organism; critical threshold temperatures (T_c_) where the mitochondrial metabolism transforms to an anaerobic mode (Reference 28). The specific growth rate (SGR) data are cited from previous studies (references 16, 18), and the temperature lethal to 50% of the samples (TL_50_) after 2 h exposure was cited from reference 19.

The limits of ventilatory and circulatory capacity are shown here to limit thermal tolerance of the sea cucumber. The appropriate temperature for growth is at 12–21°C, and the optimum temperature is at 15–18°C (Figure 4) [18]. When water temperature is over 24°C, growth performance of the sea cucumber decreased significantly [16]. At 26°C, the oxygen consumption rate increased dramatically, indicating high requirements of oxygen supply and this was associated with a rapid increase in expression of *hsp*s as the temperature increased from 26–30°C. Rapid expression of *hsp*s suggests increased hardiness against extreme thermal event and also correlates with the improved ability to recover from heat stress [23]. However, the protection is limited, and mortality of sea cucumbers occurs when temperatures exceed 30°C [19]. These results support the theory that the thermal-tolerance windows of the sea cucumber is determined by limiting aerobic scope as described by previous studies [23], [28]. The OCLTT is, therefore, an appropriate model to analyze the thermotolerance of this intertidal-subtidal species, which experiences significant daily and seasonal temperature variations in its natural habitat.

Physiological performance of organisms to temperature reflects their evolutional adaptation to local environments, and also an organism's ability to cope with climate change related temperature changes [4]. The OCLTT provides a direct link between thermal physiology and ecology of animals, which elucidates the role of aerobic metabolism in the thermal tolerance of many aquatic animals like molluscs [29] and fish across latitudes [23], [28].

In contrast to the prediction of the universal temperature-dependence model (UTD) model [8], however, the metabolic rate of a high intertidal species, *Echinolittorina malaccana,* was often negatively related to temperature over a benign range (30–40°C), and this lowering of metabolism when heated should improve energy conservation for species living in a high-temperature and food limited environment [30]. *E. malaccana* lives in the upper limit of the intertidal zone and faces dramatic temperature variation and frequent food limitation. The sea cucumber *Apostichopus japonicus* lives in the transition between the intertidal and subtidal zone, which is an important ecological boundary. The temperature variation which sea cucumbers face is not as harsh as those experienced by *E. malaccana*, and adequate food supply can be found in the habitat. Therefore, the physiologically-related thermotolerance of the sea cucumber should be an adaptation to its local environment.

## Materials and Methods

### Animals and acclimation conditions

Sea cucumbers (wet weight 10.43±0.49 g, mean± S.D.), were collected from an aquaculture facility in Jimo Town, Qingdao, China (36°21’58’’ N, 120°40’26’’ E), and were raised at 16°C for 20 days with continuous aeration. During acclimation, sea cucumbers were fed *ad libitum* daily at 14:00 using a commercial formulated feed (22.9±0.2% crude protein, 2.1±0.0% fat, 34.7±0.6% ash and 9.0±0.0% moisture, 10.6±0.0 kJ g^−1^ energy; Liuhe Aquatic feed Company, Shandong, China), containing powdered *Sargassum* spp., fish meal, sea mud, wheat, vitamin and mineral premixes. A simulated natural photoperiod cycle of 12 h light: 12 h dark was used. Seawater (30–32 psu) was sand filtered and one-half of the rearing seawater in the experiment tanks (450×250×350 mm) was changed daily.

### Metabolic performance

The metabolic performance of the sea cucumbers was assessed from oxygen consumption rate (OCR). Prior to testing, sea cucumbers were starved for 24 h to reduce associated metabolic responses. Individual sea cucumbers (wet weight  =  11.42±0.37 g) were then placed into a 330 ml conical flasks with a rubber plug, which were immersed into a water bath (Shuniu, Chengdu, China) for temperature control. To simulate the summer temperature variation in the natural habitat, water temperature in the water bath was increased from 16°C to the designated temperatures (24, 26, 28 and 30°C) at an interval of 8 h. After being maintained at the designated temperatures for 4 h, temperature was decreased gradually to 16°C within 8 h and was kept at 16°C for 4 h (Figure S5). In 16°C treatment, there were three replicates (n  =  3) and two blank controls to correct for respiration of bacteria in the water. In the four temperature variation treatments, there were five replicates (n = 5) and two blank controls. The dissolved oxygen concentrations in the bottles were measured every 2 h using a dissolved oxygen analyzer (YSI 5000, YSI, Yellow Springs, OH, USA), and the oxygen consumption rate (OCR) of the sea cucumber was calculated as [31]:
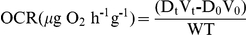
where D_t_, changes of the oxygen content (µg O_2_ L^−1^) before and after test in the test bottles; D_0_, changes of the oxygen content (µg O_2_ L^−1^) before and after test in the blank bottles; V_t_, volumes of the test bottles (L); V_0_, volumes of the blank bottles (L); W, wet weight of the sea cucumber (g); T, time duration (h).

### Heat shock response

Heat shock response (HSR) was assessed from *hsp70*, *hsp90a* and *hsp90b* (the three sequences were selected from the *A. japonicus* cDNA library from NCBI,Ajhsp70, GH985449; Aj90-a, JF907619; Aj90-b, GH550976). A total of 160 sea cucumbers (wet weight  = 10.08±0.57 g) were selected randomly and divided into four groups (40 individuals each). Water temperature was increased from 16°C to the designated temperature (24°C, F24; 26°C, F26; 28°C, F28; 30°C, F30, 5) within 8 h. After being maintained at the maximum temperatures for 4 h, water temperature was decreased to 16°C within 8 h and finally kept at 16°C for 4 h. At selected time points (6:00, 14:00, 18:00, 2:00 the next day, and 6:00 the next day), five individuals were randomly collected and their intestine, respiratory tree and body wall were dissected and stored at −80°C.

Total RNA was isolated from ∼ 80 mg of body wall, 50 mg of intestine and 50 mg of respiratory trees using Trizol Reagent (Invitrogen, Carlsbad, CA, USA). The first strand of cDNA was synthesized by using 3 µg of total RNA as a template. Partial of the sea cucumber β-actin gene (312 bp) was selected as reference to normalize the level of expression between the samples amplified using the primers from Meng et al. [25]. Primers of the four genes *hsp70*, *hsp90-a*, *hsp90-b* (Hsp70-F and Hsp70-R, Hsp90-a-F and Hsp90-a-R, Hsp90-b-F and Hsp90-b-R) were designed base on the sequences from Genbank (Ajhsp70, GH985449; Aj90-a, HO054976; Aj90-b, GH550976, Table S2).

Equal amounts of cDNA template were used in Semi-quantitative PCR. PCR conditions and components for *hsp*s and β-actin were optimized, especially for the amplification cycles and annealing temperatures. PCR was carried out in 25 µl reactions containing 2.5 µl of 10 × PCR buffer, 1.6 µl of MgCl_2_ (25 mmol l^−1^), 2.0 µl of dNTPs (2.5 mmol l^−1^), 1 µl of each primer (10 pmol ml^−1^), 15.875 µl of PCR-grade water, 0.125 µl (5U μl^−1^) of Taq DNA polymerase (Promega, Madison, WI, USA), and 1 µl of cDNA reaction mix. The programs were preceded by initial denaturation for 5 min at 94°C, followed by 30 cycles (for *hsp70*, *hsp90a* and *hsp90b*) or 28 cycles (for β-actin) of 94°C for 45 s, 53°C (for *hsp70*, *hsp90a* and *hsp90b*) or 55°C (for β-actin) for 45 s, 72°C for 1 min and a final extension step at 72°C for 10 min. All PCR products were electrophoresed in 1.2% agarose gels and stained with ethidium bromide (EB), after which the products were purified from the gel and sequenced to confirm the specificity of RT-PCR amplification. Electrophoretic images and the optical densities of amplified bands were analyzed using GeneTools software (Syngene, Frederick, MD, USA). The abundance of *hsp*s was normalized to the corresponding β-actin abundance in all samples, and expressed as the ratio optical densities of *hsp*s and β-actin (C*_hsp70_*/C_β-actin_, C*_hsp90a_*/C_β-actin_ or C*_hsp90b_*/C_β-actin_).

### Statistics

The data were analyzed using SPSS for Windows (Version 16.0; SPSS, Chicago, IL, USA). The homogeneity of variances of data was tested using Mauchly’s Test of sphericity. To investigate temporal variation in physiological performances at different temperature treatments, the oxygen consumption rate (OCR) and expression of *hsp*s among different time points and temperature treatments were analyzed using two-way ANOVA. The differences of the peak values of *hsp*s among different temperature treatments were analyzed using one-way ANOVA. The regressions of *hsp*s at the maximum temperatures (T_max_) to temperature were fitted in segmental linear regression using Prism (Version 5.0, GraphPad Software, La Jolla, CA, USA). Differences were considered significant if *P*<0.05.

## Supporting Information

Figure S1 Oxygen consumption rate of the four temperature-fluctuation (24°C, 26°C, 28°C and 30°C) treatments and the constant temperature (16°C) treatment during a 24-hour cycle in the sea cucumber *Apostichopus japonicus.*
(TIF)

Figure S2 Relative *Apostichopus japonicus* Hsp70 mRNA expression during the four temperature-fluctuation treatments in (a) intestine, (b) respiratory trees and (c) body wall.
(TIF)

Figure S3 Relative *Apostichopus japonicus hsp90_a_* mRNA expression during the four temperature-fluctuation treatments in (a) intestine, (b) respiratory trees and (c) body wall.
(TIF)

Figure S4 Relative *Apostichopus japonicus hsp90_b_* mRNA expression during the four temperature-fluctuation treatments (a) intestine, (b) respiratory trees and (c) body wall.
(TIF)

Figure S5 Diagram of the diel temperature fluctuating mode. The T_max_ of the four temperature-fluctuating treatments were 24, 26, 28 and 30°C respectively. Photoperiod regime is depicted by horizontal white (light period) and black (dark period) bars.
(TIF)

Table S1 The two-stage segmental regression of the peak values of *hsp70* and temperature in the sea cucumber, *Apostichopus japonicus.*
(DOC)

Table S2 Primer sets designed for semi-quantitative RT-PCR analysis of *hsps* mRNA in sea cucumber *Apostichopus japonicus.*
(DOC)
