# Supplementary figures and images for: Physiological Responses in a Variable Environment: Relationships between Metabolism, Hsp and Thermotolerance in an Intertidal-Subtidal Species
Source: PLoS One. 2011 Oct 17;6(10):e26446. doi: 10.1371/journal.pone.0026446 (PMC3195708; doi:10.1371/journal.pone.0026446)

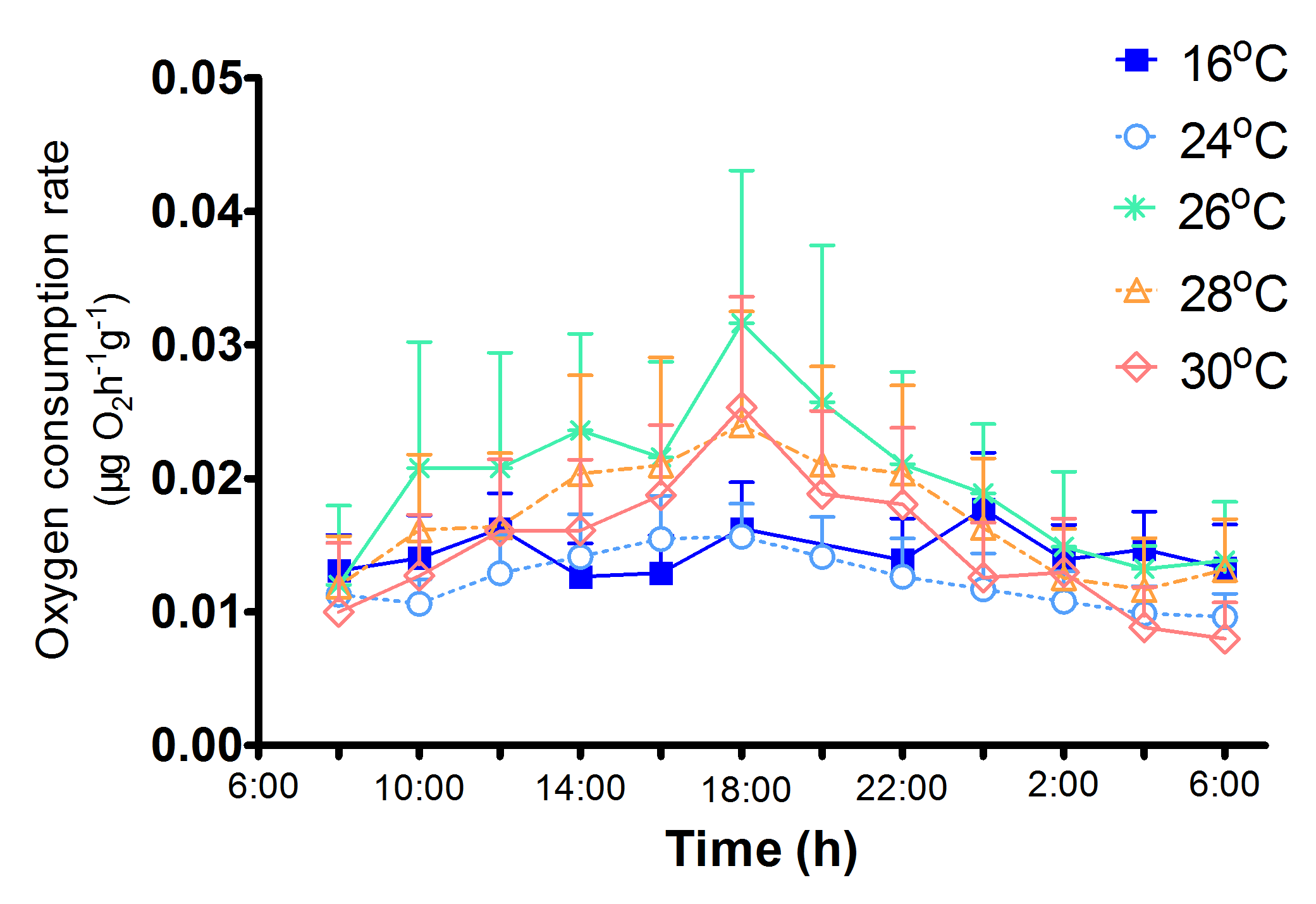

Supplement: Figure S1 — Oxygen consumption rate of the four temperature-fluctuation (24°C, 26°C, 28°C and 30°C) treatments and the constant temperature (16°C) treatment during a 24-hour cycle in the sea cucumber Apostichopus japonicus. (TIF) [file pone.0026446.s001.tif]

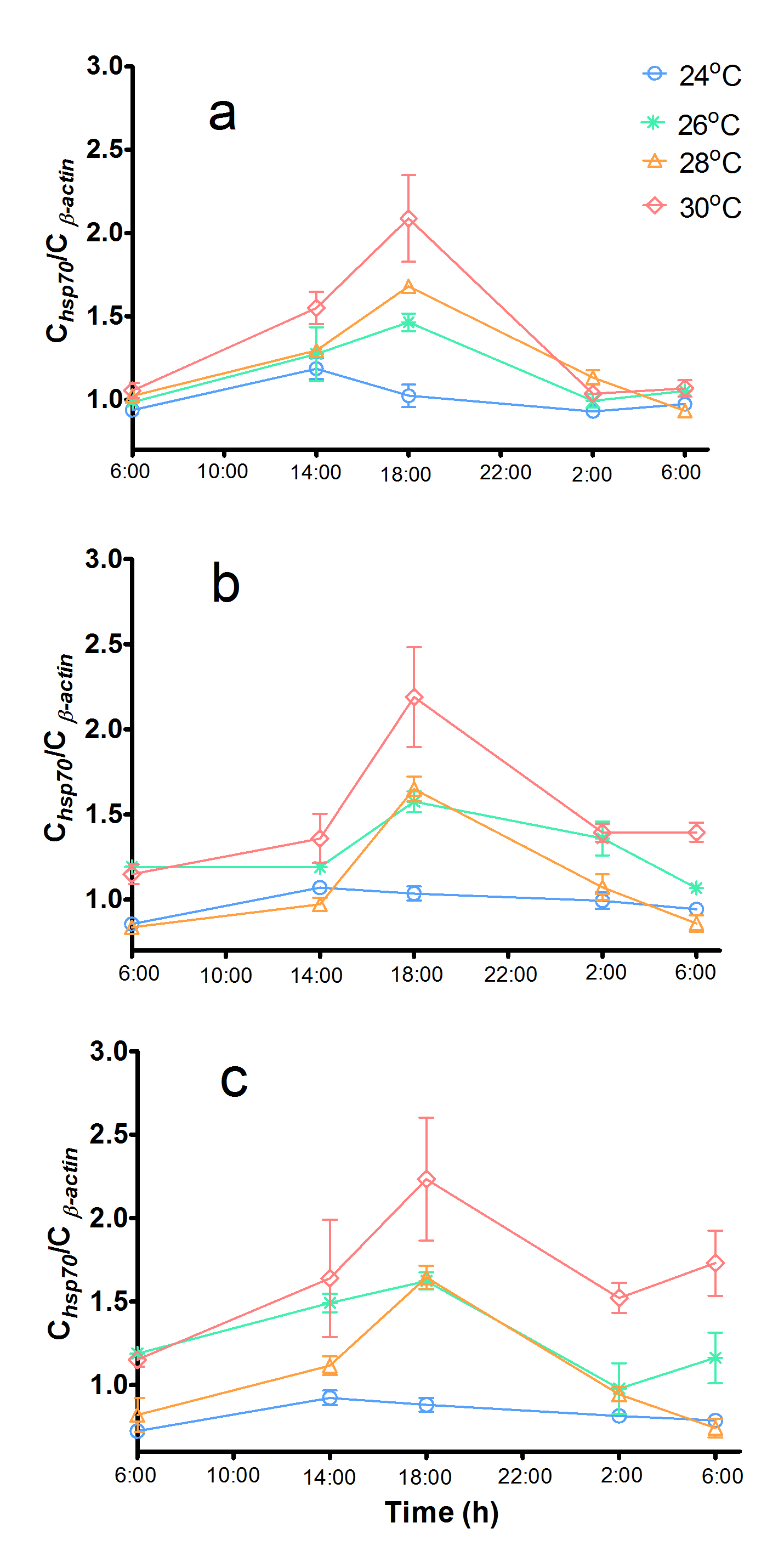

Supplement: Figure S2 — Relative Apostichopus japonicus Hsp70 mRNA expression during the four temperature-fluctuation treatments in (a) intestine, (b) respiratory trees and (c) body wall. (TIF) [file pone.0026446.s002.tif]

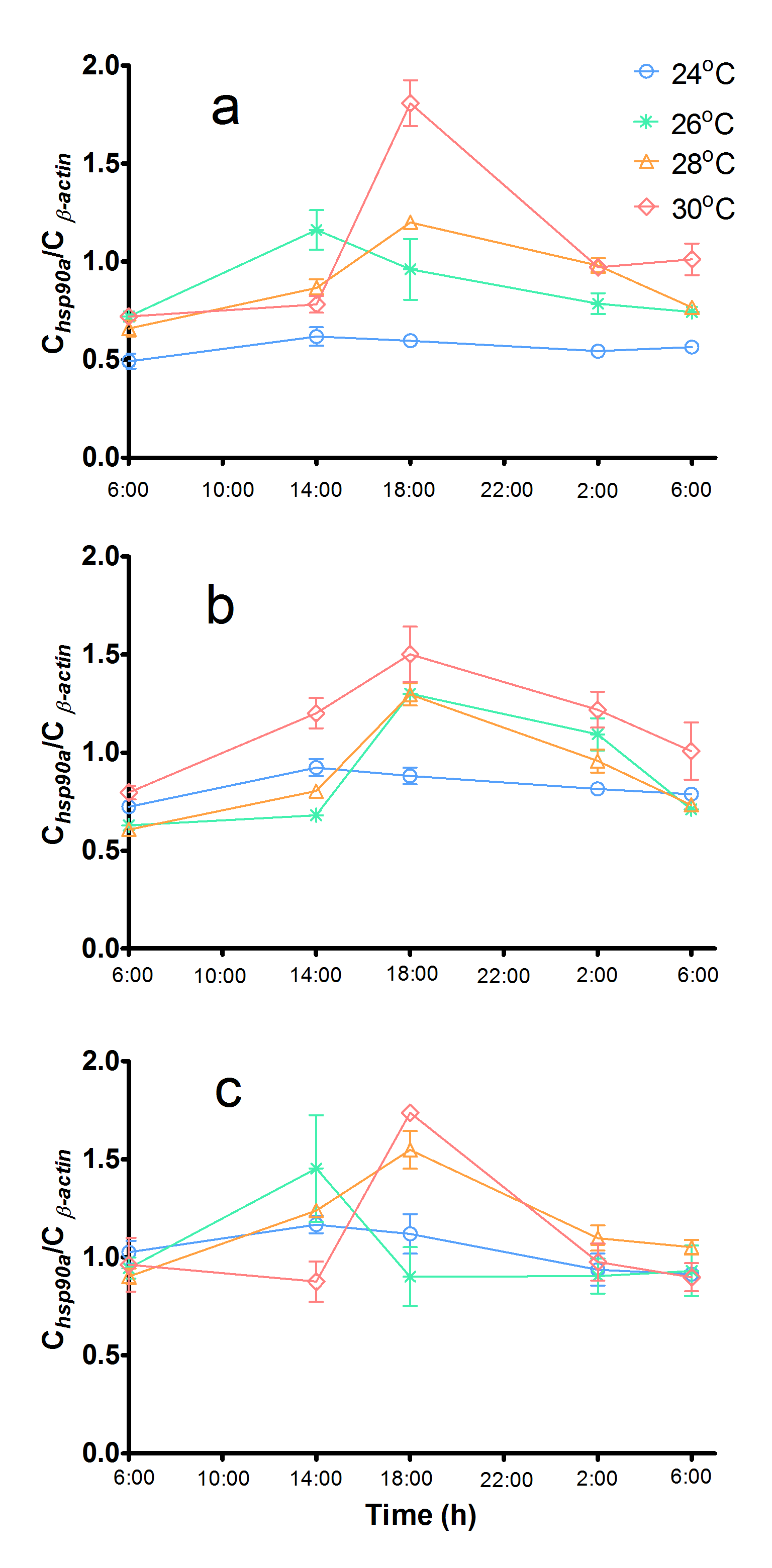

Supplement: Figure S3 — Relative Apostichopus japonicus hsp90a mRNA expression during the four temperature-fluctuation treatments in (a) intestine, (b) respiratory trees and (c) body wall. (TIF) [file pone.0026446.s003.tif]

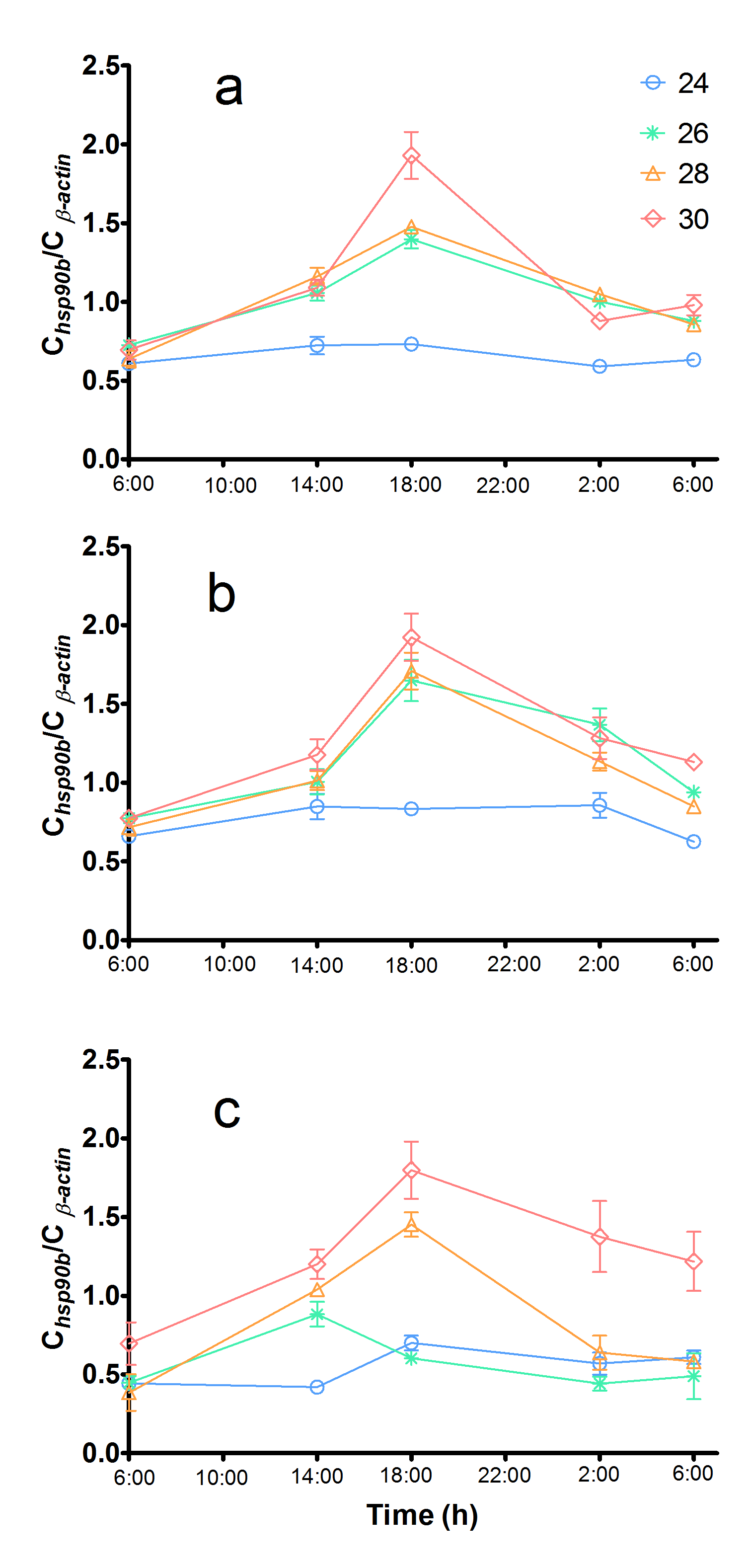

Supplement: Figure S4 — Relative Apostichopus japonicus hsp90b mRNA expression during the four temperature-fluctuation treatments (a) intestine, (b) respiratory trees and (c) body wall. (TIF) [file pone.0026446.s004.tif]

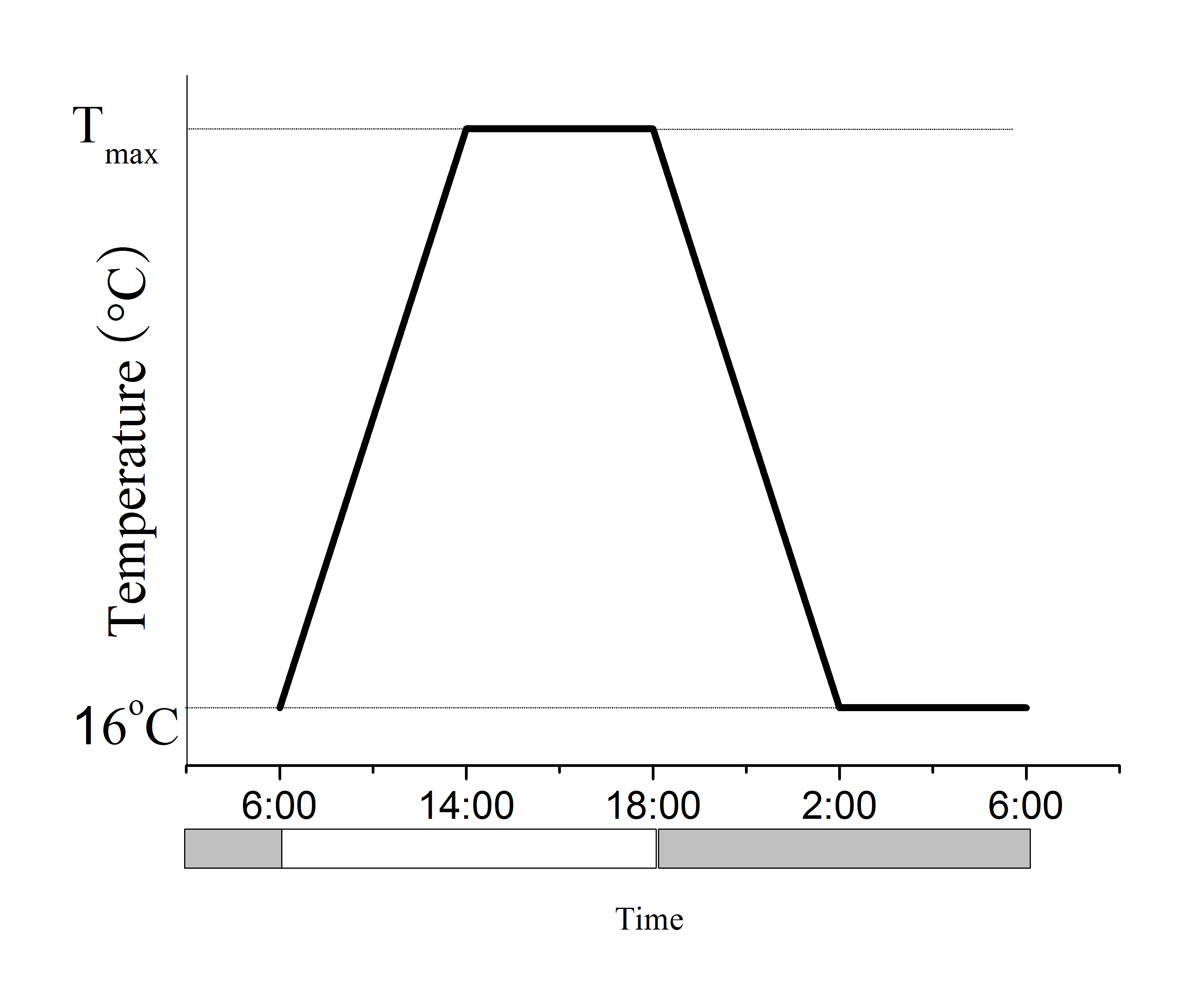

Supplement: Figure S5 — Diagram of the diel temperature fluctuating mode. The Tmax of the four temperature-fluctuating treatments were 24, 26, 28 and 30°C respectively. Photoperiod regime is depicted by horizontal white (light period) and black (dark period) bars. (TIF) [file pone.0026446.s005.tif]
