# Supplementary material for: Physiological Responses in a Variable Environment: Relationships between Metabolism, Hsp and Thermotolerance in an Intertidal-Subtidal Species
Source: PLoS One. 2011 Oct 17;6(10):e26446. doi: 10.1371/journal.pone.0026446 (PMC3195708; doi:10.1371/journal.pone.0026446)
Supplement: Table S1 — The two-stage segmental regression of the peak values of hsp70 and temperature in the sea cucumber, Apostichopus japonicus. (DOC) [file pone.0026446.s006.doc]

***Table S1*** *The two stages segmental regression of the peak values of hsp70 and temperature in the sea cucumber, Apostichopus japonicus*.

| Molecular marker | tissues | Slope 1 | Slope 2 | R^2^ |
| --- | --- | --- | --- | --- |
| *Hsp70* | Intestine | 0.007 | 0.1706 | 0.8397 |
|  | Respiratory tree | 0.023 | 0.031 | 0.7946 |
|  | Body wall | 0.002 | 0.204 | 0.7503 |
| *Hsp90a* | Intestine | -0.011 | 0.194 | 0.8988 |
|  | Respiratory tree | 0.035 | 0.093 | 0.8383 |
|  | Body wall | 0.007 | 0.170 | 0.6776 |
| *Hsp90b* | Intestine | 0.016 | 0.022 | 0.9067 |
|  | Respiratory tree | 0.023 | 0.032 | 0.8148 |
|  | Body wall | 0.021 | 0.292 | 0.9114 |
