# Supplementary material for: Physiological Responses in a Variable Environment: Relationships between Metabolism, Hsp and Thermotolerance in an Intertidal-Subtidal Species
Source: PLoS One. 2011 Oct 17;6(10):e26446. doi: 10.1371/journal.pone.0026446 (PMC3195708; doi:10.1371/journal.pone.0026446)
Supplement: Table S2 — Primer sets designed for semi-quantitative RT-PCR analysis of hsps mRNA in sea cucumber Apostichopus japonicus. (DOC) [file pone.0026446.s007.doc]

**Table S2** Primer sets designed for semi-quantitative RT-PCR analysis of *hsps* mRNA in sea cucumber *Apostichopus japonicus*1.

| Primers | Primer sequences |
| --- | --- |
| Hsp70-F | 5'-ATGCCTAGAACCAGTAGAGAAAG-3' |
| Hsp70-R | 5'-TGTCGTTCGTGATGGTGATT-3' |
| Hsp90a-F | 5'-TTGTTGAAAGGGAGGAGG-3' |
| Hsp90a-R | 5'-GGCATCAGAGGCGTTAGA-3' |
| Hsp90b-F | 5'-TCTTTCTTAGGGAACTCATCTC-3' |
| Hsp90b-R | 5'-CCTGTAGCATTCGTCATCG-3' |
| β-actin-F | 5’-ACACGGTATCGTCACAAACTGG-3' |
| β-actin-R | 5'-AGGATAGCGTGAGGAAGAGCAT-3' |

^1^Hsp70-F and Hsp70-R are used for amplifying *hsp70*, Hsp90a-F and Hsp90a-R are used for amplifying *hsp90a*, Hsp90b-F and Hsp90b-R are used for amplifying *hsp90b*, and β-actin-F and β-actin-R are used for amplifying β-actin.
